# Colorectal Neoplasia Pathogenesis in Normal Appearing Colonic Mucosa ‐A Perspective With Focus on Eicosanoid Signaling Pathways

**DOI:** 10.1002/cam4.71168

**Published:** 2025-09-22

**Authors:** Ulrike Ries Feddersen, Sebastian Kjærgaard Hendel, Victoria Hellen Berner‐Hansen, Simon Veedfald, Mark Berner‐Hansen, Niels Bindslev

**Affiliations:** ^1^ Digestive Disease Center Bispebjerg Hospital, University of Copenhagen Copenhagen Denmark; ^2^ Gastrounit, Surgical Section Hvidovre University Hospital Copenhagen Denmark; ^3^ Department of Radiology Zealand University Hospital Roskilde Denmark; ^4^ Zealand Pharma Copenhagen Denmark; ^5^ Department of Biomedical Sciences University of Copenhagen Copenhagen Denmark

## Abstract

Colorectal cancer (CRC) is a major cause of cancer‐related mortality, especially in the Western world, and its incidence is expected to increase in the years to come. Prevention and early detection are key strategies to improve CRC morbidity and mortality. Although pathogenesis is still not fully understood, several signaling pathways have been studied and some are associated with the development of colorectal neoplasia (CRN) and CRC. Further identification of individuals with an increased risk of developing CRN would allow optimization of surveillance programs and help guide pharmacological preventive strategies. This perspective review outlines signaling pathways, biomarkers, and related pharmacological targets potentially implicated in the pathogenesis of CRN. We present our research based on studies carried out in normal appearing colonic mucosa from patients with and without CRN. With a focus on arachidonic acid signaling pathways, and in contrast to many other studies on cell culture and CRN tissue samples, our research is based on fresh colonic biopsies from normal tissue and presents and documents alterations in the function, expression, and location of enzymes, receptors, and transporters potentially involved in CRN pathogenesis. Based on these findings, we suggest areas of focus for future research and drug development for prevention and maybe even treatment of CRN and CRC. Furthermore, based on our observations of the COX‐1 enzyme, we also discuss the implications of this enzyme in the development of CRN.

**Abbreviations**
15‐PDGH: 15‐prostaglandin dehydrogenase
AA: arachidonic acid
ABC: transporter
ACh: acetylcholine
ALA: alpha‐linolenic acid
ASA: acetylsalicylic acid
ATP: binding‐cassette transporter
BChE: butyrylcholinesterase
cAMP: cyclic adenosine monophosphate
cGMP: cyclic guanosine monophosphate
CN: cyclic nucleotide
COX‐1: cyclooxygenase 1
COX‐2: cyclooxygenase 2
CRC: colorectal cancer
CRN: colorectal neoplasia
CTLs: choline transporter‐like proteins
CYP450: cytochrome P450
DHA: docosahexaenoic
ELISA: enzyme‐linked immunosorbent assay
EP1‐4: prostaglandin subtype receptors 1‐4
EPA: eicosapentaenoic
ETA: eicosatetraenoic
IHC: immunohistochemistry
LA: linoleic acid
LOX: lipoxygenases LOX‐5, LOX‐12, and LOX‐15
M2: muscarinic receptor type 2 (M2)
MUAS‐Chamber: modified Ussing air suction chamber
NSAID: nonsteroid anti‐inflammatory drug
OATP: organic‐anion transporter protein
PDE: phosphodiesterase
PGE_2_
: prostaglandin E2
PGT: prostaglandin transporter
PLA2: phospholipase A2
PLA2G4A: cytoplasmic phospholipase 2A
PTGES: prostaglandin E synthase
PTGIS: prostaglandin I synthase
qPCR: quantitative real time polymerase chain reaction
SCC: short circuit current
WB: Western blot

## 1 Introduction

Colorectal cancer (CRC) is one of the most devastating cancers worldwide in terms of mortality and incidence [1, 2]. Despite extensive research, the underlying pathogenic mechanisms for CRC are still not fully understood. Current research from cell cultures and studies of premalignant and malignant colorectal tissues suggests that benign colorectal neoplasia (CRN), primarily adenomas, can transform into CRC in the form of adenocarcinomas. This transformation is attributed to the accumulation of mutations and chromosomal deletions [3, 4].

**FIGURE 1:**
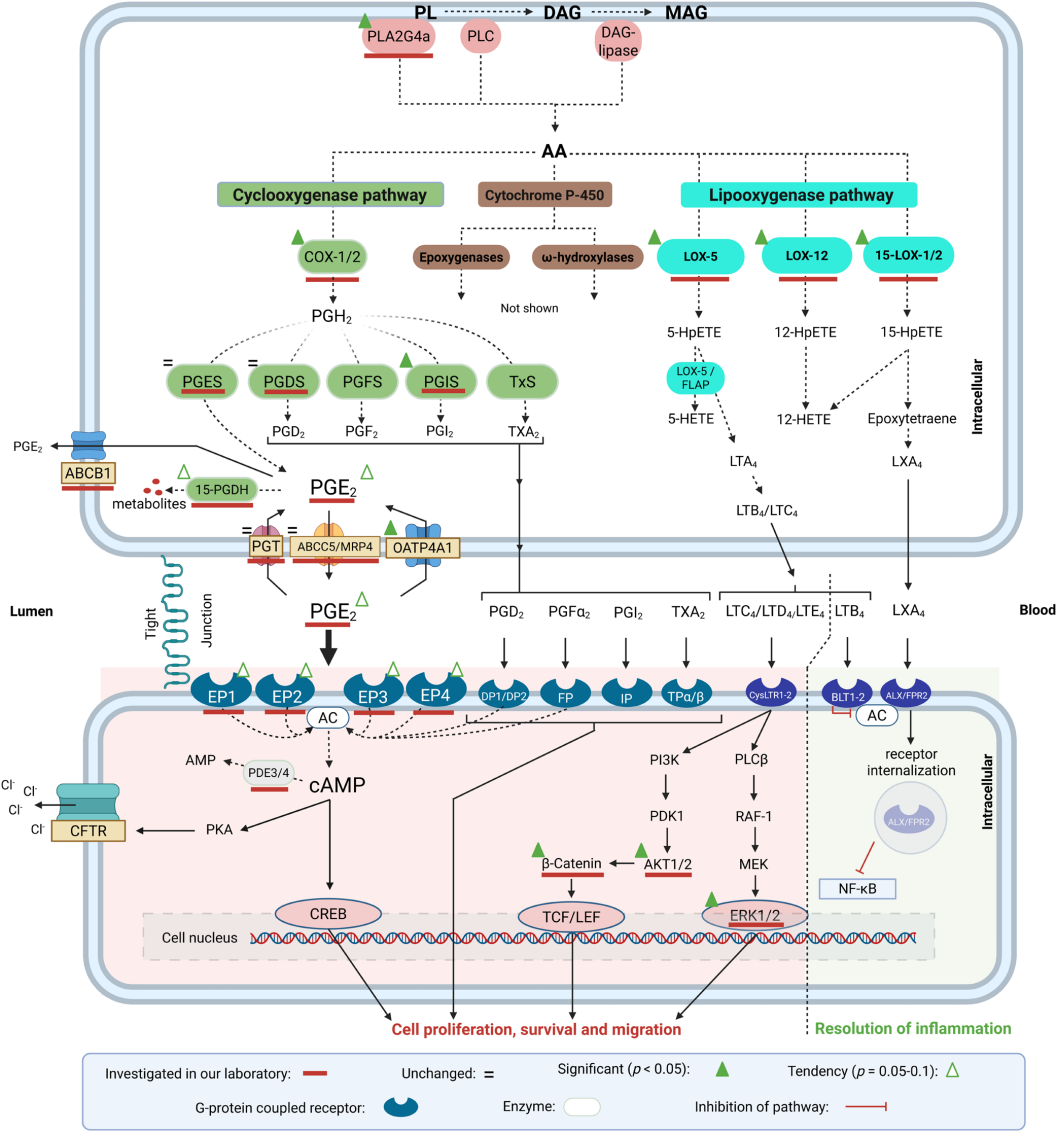
Arachidonic acid metabolization, its products and downstream signaling (not all pathways are shown). **Upper cell:** Arachidonic acid (AA) is released from the cell membrane by either the enzyme phospholipase A2 (PLA2G4a) which cleaves AA from membrane phospholipids (PL), or by phospholipase C (PLC) converting PL to diacylglycerol (DAG), which is further converted to monoacylglycerol (MAG) and free AA by diacylglycerol‐lipase (DAG‐lipase). AA is then further metabolized by (1) cyclooxygenases (COX‐1 and COX‐2), (2) Lipoxygenases (LOX) comprising three main enzymes: 5‐LOX, 12‐LOX and 15‐LOX (15‐LOX‐1 and 15‐LOX‐2), and (3) Epoxygenases and ω‐hydroxylases. In the COX‐pathway AA is metabolized into prostaglandin H2 (PGH_2_) and further into other prostaglandins: PGE_2_, PGD_2_, PGF_2_, PGI_2_ and thromboxane A_2_ (TXA_2_) by different synthases. PGE_2_ is degraded by 15‐prostaglandin dehydrogenase (15‐PGDH). LOX enzymes metabolize AA into hydroperoxyl‐eicosatetraenoic acids (HPETEs) and further to hydroxyeicosatetraenioc acids (HETEs). 5‐LOX‐activating protein (FLAP) also metabolizes 5‐HpETE into leukotriene A_4_ (LTA_4_), which is further converted into leukotriene B_4_, ‐C_4_ and (LTB_4_, ‐C_4_). These products are transported to the extracellular space by different transporters (only PGE_2_ transporters shown), where LTC_4_ is transformed into LTD_4_ and LTE_4_. Here, they all bind to and activate their respective receptors. **Lower cell:** The red area in the lower cell indicates pathways shown that promote cell proliferation and survival, whereas the green area shows pathways promoting resolution of inflammation. **RED:** When activated by PGE_2_, EP‐receptors mediate the conversion of ATP to cAMP via the membrane bound enzyme adenylate cyclase (AC). cAMP promotes proliferative properties through activation of the transcription factor “cAMP response element‐binding protein” (CREB) and stimulates luminal chloride secretion. Phosphodiesterases (PDEs) converts cAMP to AMP. Other prostaglandins and TXA_2_ also stimulate cell proliferation and survival, some via AC activation, others by mechanisms not shown. LTC_4_, LTD_4_ and LTE_4_ bind and activate CysLTR‐1 and ‐2 initiating various downstream activities, for example, mitogen‐activated protein kinase (MAPK)/extracellular signal‐regulated kinase (Erk) pathways, and PI3K/Akt pathway where PI3K generates PIP3 and further in association with PDK1 activates Akt. Akt excretes its functions through modulation of downstream substrates such as mTOR and β‐catenin. Cytoplasmic β‐catenin translocate to the nucleus and interacts with T‐cell factor/lymphoid enhancer factor (TCF/LEF), and thus activating an array of regulatory genes. **GREEN:** Conversely, activation of FPR2/ALX and BLT1‐2 inhibits proinflammatory pathways signaling and thus promote resolution of inflammation. **How to read the figure:** A red bar indicates receptors, enzymes and related molecules investigated in our laboratory. Others illustrated are not investigated but are relevant for future research and general apprehension. A full green triangle means an observed increase, an empty green triangle indicates an observed tendency (*p* = 0.05–0.1), and an equals sign indicates no observed deviation. These indicators are based on an overall assessment across different studies and methods. Elaborated, based on Feddersen et al. [9]. Created in BioRender.com. Kjaergaard (2021) BioRender.com/s45c847.

Known risk factors for CRN and CRC include genetic predispositions and chronic colorectal inflammation induced by eicosanoid lipids [5, 6]. One such lipid is arachidonic acid (AA) canonically released from cell membrane phospholipids by phospholipase A2 (PLA_2_) [7, 8]. The free AA is metabolized by several enzymes, such as cyclooxygenases (COXs), lipoxygenases (LOXs), and cytochrome P450 (CYP450) resulting in a host of different metabolites as signaling molecules, which work through separate receptors and pathways, Figure 1 [10]. Prostaglandin E2 (PGE_2_) is an AA signaling metabolite produced together with other prostaglandins by two subtype COX enzymes: cyclooxygenase 1 (COX‐1) and cyclooxygenase 2 (COX‐2). Increased expression of especially the “inducible” COX‐2 isoform and augmented PGE_2_ levels are linked to a higher risk of developing CRC, as PGE_2_ induces cell proliferation, migration, and survival by binding to its surface receptors (EP1‐4), Figure 1 [7]. Interestingly, treatment with the nonsteroid anti‐inflammatory drug (NSAID) aspirin, which inhibits both COX‐1 and COX‐2, seems to reduce the risk of developing CRC [11, 12]. Meanwhile, the “constitutive” subtype COX‐1 is less studied, likely due to a common “misconception” that it is solely responsible for maintaining homeostatic conditions.

To spot early signs of aberrant expression and function in AA signaling pathways, which could be helpful in the search of markers for the prevention and treatment of CRC, our research has focused on investigating macroscopically normal appearing colonic mucosa from patients with current or previous CRN as compared to individuals with no history of CRN. The rationale of this approach has been to identify individuals with early mucosal changes toward CRN and CRC.

This perspective review presents some of our research into CRN‐pathogenesis. We focus on observations only from normal appearing colonic mucosa, and mainly on the COX‐1/COX‐2‐PGE_2_ signaling pathway and its related enzymes, receptors, and transporters. Some data from the LOX and acetylcholine (ACh) pathways are also included. We discuss how these findings may be useful in the development of biomarkers for early disease detection of CRC and possible perspectives for preventive and therapeutic drug development. Furthermore, we explore the potential role of both COX‐1 and COX‐2 in the CRN and CRC pathogenesis and discuss how the importance of COX‐1 might have been overlooked.

## 2 Experimental Approach

Our multifaceted investigational approach in terms of patient population and methods as well as key findings are summarized in Table 1. A brief description is given of our *modified Ussing air suction chamber* (MUAS‐chamber) functional method for measuring tissue activity as expressed by short‐circuit current (SCC) [19]. For a detailed account of the remaining applied modalities, that is, quantitative real time polymerase chain reaction (qPCR), western blot (WB), immunohistochemistry (IHC), and enzyme‐linked immunosorbent assay (ELISA), we refer to the original papers, table 1 [9, 13, 14, 15, 16, 17, 18].

**TABLE 1:** Paper overview.

Publication author & reference	*N*	Methods	Key findings & conclusions
Kaltoft et al. [13]	63	MUAS, Histology	CRN patients express higher COX enzyme activity Stimulation of EP4 receptor elicit largest response
Kleberg et al. [14]	19	MUAS, qPCR, IHC, Histology	PGE_2_ influx transporters upregulated in CRN patients CRN patients demonstrate reduced transport capacity to db‐cAMP
Mahmood et al. [15]	27	MUAS, qPCR, IHC	Activity and expression of PDE4B and PDE5A are increased in CRN patients
Damm et al. [16]	42	MUAS, qPCR, IHC	Baseline SCC is higher in CRN patients, indication a prostaglandin‐induced lifted anion secretion mRNA levels of choline transporter‐like proteins 1 and 4 are increased in CRN patients
Jensen et al. [17]	43	MUAS, qPCR, IHC	The combined activity of COX‐1 and COX‐2 is increased in CRNs Epithelial COX‐1 expressing cells are tuft cells
Petersen et al. [18]	27	qPCR, IHC, WB, ELISA	Several PGE_2_‐related genes are up‐regulated in CRN patients ERK1 may prove useful as a predictive biomarker for CRN development
Feddersen et al. [9]	73	MUAS, qPCR	Several PGE_2_‐related genes are up‐regulated in CRN patients PGE_2_ binds to a high‐ and a low affinity receptor with a sensitivity in the nanomolar range The EP4 receptor demonstrates the highest secretory response

*Note:* Annotated overview of studies published from our laboratory. Number of patients included, methods used, and main conclusions are shown.

Abbreviations: COX, cyclooxygenase; CRN, colorectal neoplasia; CTRL, controls; db‐cAMP, di‐butyryl‐cyclic adenosine monophosphate; ELISA, enzyme‐linked immunosorbent assay; EP4, prostaglandin E2 receptor 4; ERK1, extracellular signal‐regulated kinase 1; IHC, immunohistochemistry; MUAS, modified ussing air suction; PDE, phosphodiesterase; PGE_2_, prostaglandin E2; qPCR, quantitative real time polymerase chain reaction; SCC, short circuit current; WB, western blot.

### 2.1 Patient Populations

Almost 300 patients were included in the seven studies, and the main conclusions of these studies are presented in Table 1 [9, 13, 14, 15, 16, 17, 18]. The patients were divided into two groups based on their medical history and endoscopic evaluation: individuals with a history of CRN or diagnosed with CRN at the time of endoscopy were categorized as “CRN patients”, while patients with neither a history nor a current diagnosis of CRN nor CRC served as controls, “CTRLs.” A complete colonoscopy was an inclusion criterion. CRN comprised sessile serrated lesions (all types), high‐ and low‐grade tubular adenomas, villous adenomas, tubule‐villous adenomas, as well as adenocarcinomas. For a detailed account of inclusion and exclusion criteria, we refer to the individual papers [9, 13, 14, 15, 16, 17, 18].

### 2.2 Tissue Specimens

Mucosal biopsies were obtained during colonoscopies using standard endoscopic biopsy forceps. On retraction of the endoscope, biopsies were collected 20–40 cm from the anal verge from macroscopically normal appearing colonic mucosa, and at least 10 cm from macroscopically abnormal appearing mucosa, that is, adenomas. Biopsies were either mounted in MUAS‐chambers immediately after collection or stored for pending analyses.

### 2.3 Functional Studies in MUAS‐Chambers

**FIGURE 2:**
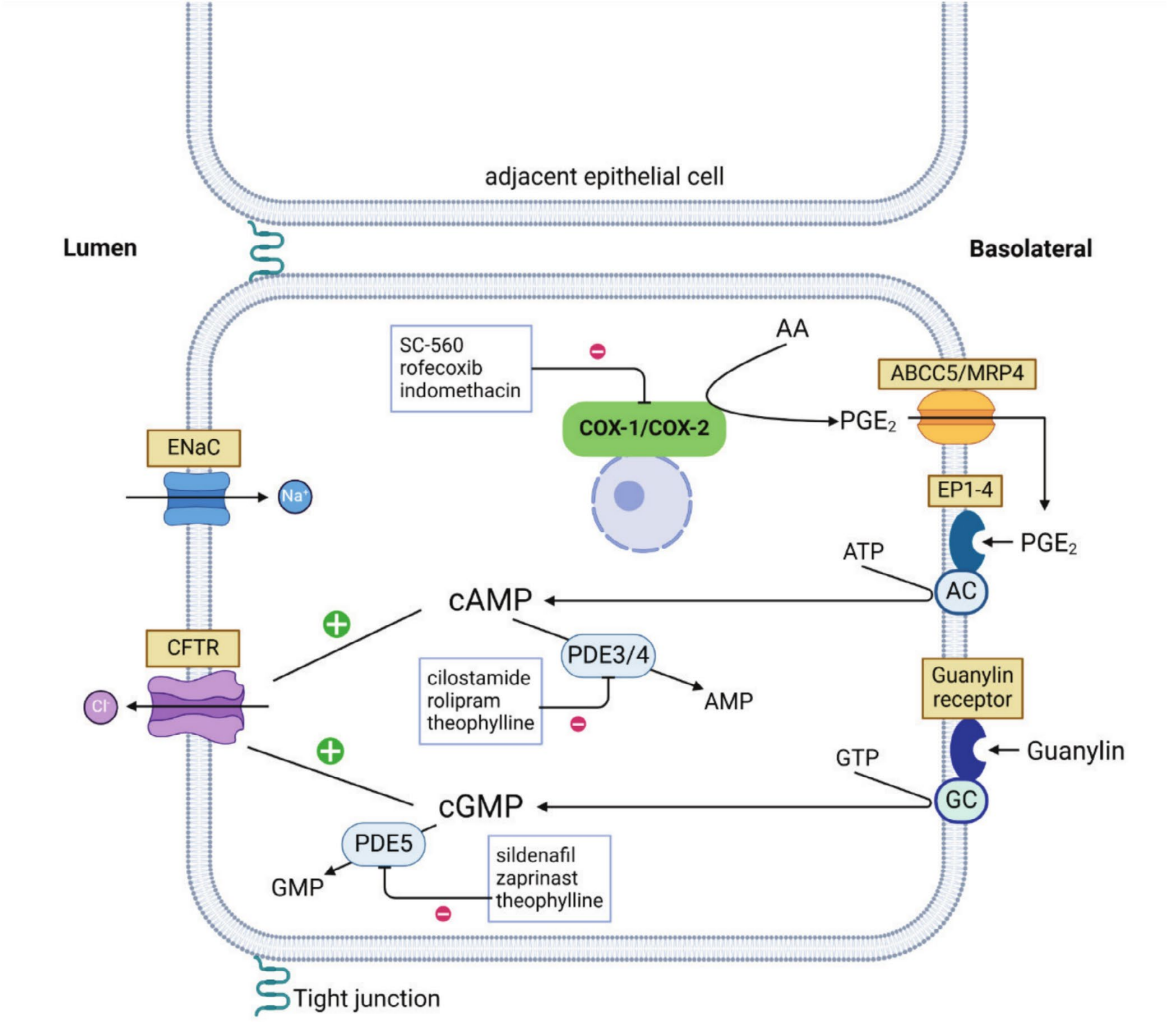
Simplified model of Cl^−^ secretory mechanisms in a colonic epithelial cell. Cyclooxygenase 1 (COX‐1) and ‐2 (COX‐2) produce PGE_2_ from free arachidonic acid (AA). COX‐1 is inhibited specifically by SC‐560 and COX‐2 by rofecoxib, while indomethacin inhibits both COX isoenzymes nonspecifically. When synthesized, PGE_2_ leaves the cell through the basolateral membrane and exerts its function either by auto‐ or paracrine stimulation. PGE_2_ binds to its receptor (EP‐receptors) and stimulates synthesis of second messenger cAMP from ATP by adenylate cyclase (AC). cGMP is synthesized from GTP through activation of a guanylin receptor and thus the guanylin cyclase (GC). Both cAMP and cGMP stimulate luminal Cl^−^ secretion and are simultaneously degraded to AMP and GMP by different phosphodiesterases (PDEs). Cilostamide and rolipram specifically inhibit PDE3 and PDE4, while sildenafil and zaprinast inhibit PDE5. Theophylline is a nonspecific PDE inhibitor. Amiloride inhibits ENaC mediated luminal sodium absorption. Modified from figure 5 in Kjaergaard et al. [23]. Created in BioRender.com. Kjaergaard (2021) BioRender.com/s45c847.

Biopsies were mounted in MUAS chambers, bathed in a D‐glucose‐supplied bicarbonate‐Ringer solution, and electrogenic mucosal ion transport properties (expressed as SCC) were measured before and after pharmacological intervention. Depending on the experiment, biopsies were treated with amiloride (epithelial sodium channel, ENaC, inhibitor), theophylline (nonspecific phosphodiesterase [PDE] inhibitor), indomethacin (a nonselective COX inhibitor), SC‐560 (selective COX‐1 inhibitor), rofecoxib (selective COX‐2 inhibitor), PGE_2_, and ACh to explore various aspects of the PGE_2_‐ and ACh‐induced secretory pathways. Experiments were terminated with the addition of bumetanide (Na^+^/K^+^/2Cl^−^cotransporter inhibitor) and/or ouabain (Na^+^/K^+^‐ATPase inhibitor) to verify the viability of the tissue, Figure 2. Chloride secretion was measured as changes in SCC after inhibition of electrogenic luminal sodium absorption. Changes in SCC due to manipulations of specific components of the PGE_2_ signaling pathway were used as a surrogate or indirect marker of enzyme, receptor, and/or transporter activity [19].

## 3 Main Findings

Here, we present our main findings related to two cyclic adenosine monophosphate (cAMP)‐dependent signaling systems, the COX‐PGE_2_ and LOX‐leukotriene (LT) pathways, involving effectors such as EP receptors, organic anion transporting polypeptide (OATP)‐, ATP‐binding cassette (ABC)‐ transporters, cyclic nucleotide PDEs, and additionally an ACh‐Ca^2+^‐pathway with related proteins.

### 3.1 COX‐PGE_2_ Pathway

#### 3.1.1 COX‐Isoenzymes

In our first study, Kaltoft et al. [13] demonstrated that the COX‐activity was elevated in colonic mucosa from CRN patients compared to CTRLs. However, the study did not distinguish between the contributions of the two COX isoenzymes. Jensen et al. [17] later clarified that the observed increase was due to an increase in both COX‐1 and COX‐2 activity, as neither isoenzyme individually showed significant changes. This suggests that both isoforms act synergistically as drivers in the CRN‐related COX‐PGE_2_ signaling cascade.

**FIGURE 3:**
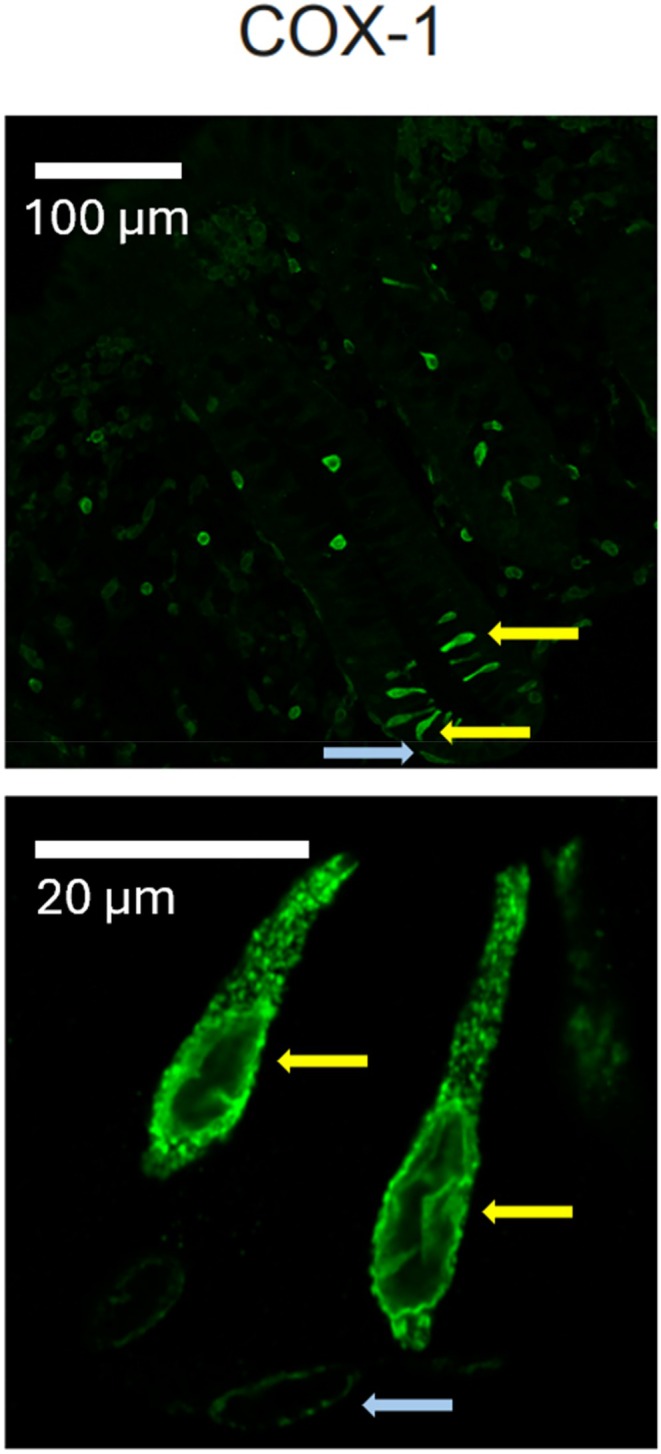
Cyclooxygenase 1 (COX‐1) staining of healthy human sigmoid colon. Yellow arrows indicate COX‐1 positive tuft cells, and the blue arrows indicate a subepithelial cell that may represent pericryptal fibroblasts described by Roulis et al. [21]. This figure is adapted from: “Possible predisposition for colorectal carcinogenesis due to altered gene expression in normal appearing mucosa from patients with colorectal neoplasia” by Petersen et al. [18].

In addition to the functional outcome, qPCR analyses were performed on relevant genes including COX‐enzymes. Upregulation of COX‐1 and COX‐2 mRNA was observed in CRN patients; however, it was not statistically significant [17]. Petersen et al. [18] later confirmed this and furthermore found a 26% higher mucosal content of active PGE_2_ in CRN patients compared to controls. To our surprise, these PGE_2_ concentrations were a factor 500 larger than the concentration of extracellular PGE_2_ necessary for eliciting a 50% stimulation of the EP4 receptor, which will be discussed below in the Perspectives section. Unexpectedly, while COX‐2 within the colonic epithelium was delimited to the cytoplasm of absorptive cells, COX‐1 was only, but markedly, present in tuft cells in colonic epithelia, Figure 3 [17]. This observation was studied subsequently in a broader context [22, 23].

#### 3.1.2 EP‐Receptor Subtypes and Downstream Gene Expression

Moving further downstream in the COX‐PGE_2_ signaling pathway, functional studies of PGE_2_ EP receptors in MUAS‐chambers were performed by exposing colonic mucosal biopsies to PGE_2_ as well as selective agonists of the four EP receptor subtypes, Figure 1 [9]. Experiments suggested that at least two types of EP receptors were activated—a high and a low affinity receptor, with EC_50_s spaced by more than 15 folds. Based on applied agonists receptor selectivity, our findings suggest activation of a high affinity EP4 receptor and a low affinity EP2 receptor [9].

We observed that both nonspecific stimulation with PGE_2_ as well as selective agonism on the EP4 receptor subtype revealed a highly potent ligand‐receptor interaction with activation even at concentrations as low as 1 nM [9, 20]. Both Petersen et al. and Feddersen et al. performed qPCR analyses on all four EP receptors and demonstrated a trend towards upregulation of all EP receptors [9, 18]. The COX‐PGE_2_ signaling pathway was further characterized by determining the expression of genes encoding proteins involved in the downstream signaling cascade of the EP receptors, Table 2 and Figure 1. Finally, the expression of ERK2, Akt1, Akt2, and PPP2R1B was increased, whereas the expression of beta‐catenin, GSK3B, and PTPRM was unchanged, Tables 1 and 2 [18]. Protein levels were analyzed with WB, but no relevant changes were detected between patient groups [18].

**TABLE 2:** Overview of genes analyzed by qPCR.

Study	Gene	*p*	Changes in CRN patients compared to CTRL
Kleberg et al. 2012	OATP1C1	NA	—
	OATP2B1	0.017*	↑
	OATP4A1	0.013*	↑
	OATP4C1	NA	—
	ABCB1	NA	—
	ABCC3	NA	—
	ABCC4	NA	—
	ABCC5	NA	—
Mahmood et al. 2016	PDE3A	NA	—
	PDE3B	NA	—
	PDE4A	NA	—
	PDE4B	0.002**	↑
	PDE4C	NA	—
	PDE4D	0.0712	—
	PDE5A	0.02*	↑
	PDE10A	0.06	—
Damm et al. 2017	CTL1	0.00018**	↑
	CTL2	0.70	—
	CTL3	0.13	—
	CTL4	0.040*	↑
	CTL5	0.80	—
	OCT1	0.24	—
	OCT2	0.41	—
	OCT3	0.66	—
	VAChT	0.21	—
	CHaT	0.98	—
	CarAT	0.67	—
	M1	0.099	—
	M2	0.44	—
	M3	0.27	—
	AChE	0.24	—
	BChE	0.10	—
Jensen et al. 2018	COX‐1	0.24	—
	COX‐2	0.43	—
Petersen et al. 2019	PLA2G4A	0.020*	↑
	COX‐1	0.0252	—
	COX‐2	0.049	—
	PTGES	0.388	—
	EP1	0.626	—
	EP2	0.207	—
	EP3	0.016*	↑
	EP4	0.096	—
	PGT	0.217	—
	15‐PGDH	0.066	—
	ERK1	0.007**	↑
	ERK2	0.020*	↑
	Akt1	0.020*	↑
	Akt2	0.041*	↑
	ß‐Catenin	0.082	—
	GSK3ß	0.054	—
	PPP2R1B	0.030*	↑
	PTPRM	0.059	—
Feddersen et al. 2022	EP1	0.03*	↑
	EP2	0.03*	↑
	EP3	0.54	—
	EP4	0.09	—
	LOX‐5	0.006**	↑
	LOX‐12	0.04*	↑
	LOX‐15	0.005**	↑
	PTGDS	0.36	—
	PTGIS	0.04*	↑
	ARK1B1	0.15	—

*Note:* Overview of investigated genes using qPCR across the studies.

Abbreviations: CRN, colorectal neoplasia; CTRL, control; NA, not available.

* *p* < 0.05.

** *p* < 0.01.

Upwards arrow: increased expression. Horizontal bar: no changes.

### 3.2 LOX‐LT Pathways

In a pilot study, mRNA expression of three key enzymes in the LOX‐LT pathway was investigated in normal appearing colon mucosa from CTRL and CRN patients [9]. Interestingly, increased mucosal expression of all enzymes: LOX‐5, LOX‐12 and LOX‐15 was found in CRN patients compared to CTRLS. Unfortunately, the employed LOX‐15 primer did not differentiate between the two LOX‐15 subtypes. However, our findings suggest a possible role of this pathway in the pathogenesis of CRN, further discussed below in the Perspectives section below [9].

### 3.3 Cyclic Nucleotide PDEs

We investigated cyclic nucleotide PDEs in normal appearing colonic mucosa, based on the hypothesis that the function of PDEs and cyclic nucleotides (CN) degradation, and thus a changed intracellular signaling through cAMP and cyclic guanosine monophosphate (cGMP) might be altered in CRN patients, as it has been observed in CRC, Figure 1 [15, 24, 25]. Functional studies by Mahmood et al. [15] revealed a lower activity of PDE4 in CRN patients, while subsequent expressional studies found a higher expression of PDE4B and PDE5A in CRN patients, Table 2. Furthermore, the expression of seven PDE isozymes in the colonic mucosa was confirmed; however, without differences between patient groups [15].

### 3.4 OATP‐ and ABC‐Transporters

A dysfunction in OATP‐ and ABC‐transporters, which impairs the excretion of for example xenobiotics, toxins, and waste metabolites, and consequently disrupts the maintenance of a healthy microenvironment, has been associated with cancer, including CRC development [7, 26, 27].

Kleberg et al. [14] aimed to functionally characterize the excretory system of the human colon by means of CNs, which are shared substrates for some OATP‐ and ABC‐transporters. In MUAS‐chambers, dibutyryl‐cAMP, one of four CN tested, induced the highest SCC in CRN as well as CTRL patients; though this effect was reduced in CRN patients. Additionally, qPCR analyses disclosed increased mRNA levels of transporters OATP4A1 and OATP2B1 in CRN patients, both able to transport basolateral PGE_2_ into columnar and endocrine epithelial cells. PGE_2_ secretion was found to occur via ABCC5 and apically through ABCB1, Figure 1 [14].

### 3.5 Acetylcholine‐Related Proteins

Epithelial transporters and metabolites related to the non‐neuronal ACh metabolism were investigated by Damm et al. [16]; see Figure 1 in the original paper. In the human colon, butyryl‐cholinesterase enzyme (BChE), an enzyme that breaks down ACh into acetate and choline, and the choline transporter‐like proteins CTL1, CTL3, and CTL4 are expressed in crypt cells and involved in the mucosal nonneuronal ACh metabolism [16]. The most predominant CTLs were CTL1 and CTL4, while CTL2 and CTL3 were less expressed. CTL5 was barely expressed. The expression of CTL1 and CTL4 genes was increased in patients with CRN, Table 2. The vesicular ACh transporter protein was similarly expressed in the two patient groups. This information is worth keeping in mind when considering causes for CRN development.

In functional MUAS chamber studies, ACh induced a fast dose‐dependent biphasic SCC response, indicating channel‐dependent ion currents and the presence of high and low affinity receptors. The muscarinic receptor type 2 (M2) showed the highest mRNA expression, followed by M1 and M3. Expression was similar in CRN and CTRL patients [16].

## 4 Perspectives

Using a multimodal approach, our research focuses on characterization of normal appearing colonic mucosa from individuals with and without a history of CRN, thus avoiding macroscopically evident neoplastic tissue. Many other studies are performed on either rodent tissue, cell lines such as the Caco‐2 cells (from human CRC) or even cancerous tissue samples. As a marker of cellular transformation and carcinogenesis, multiple reports have shown elevated levels of AA products pathways such as COX‐2‐PGE_2_, LOX, and its downstream products [28, 29, 30]. These changes are particularly pronounced in malignant tissues and cell lines, where the expression of several protein systems involved in signal transduction, such as receptors, downstream effectors, and their associated pathways, often differs markedly from that seen in normal tissue. Contrary, our experimental approach allows us to identify potential early changes in signaling pathways, which might be indicative of an emerging CRN pathogenesis. For more than a decade, we have generated evidence of changes in normal appearing colonic mucosa from CRN patients compared to CTRLs, not only in expression studies but also in terms of localization, accumulation of signaling molecules, and their elicited function. A similar approach with non‐diseased tissue samples was pursued by Polley and coworkers [31], who conducted a study of proteomics, which showed that a wide range of proteins had a modified expression in apparently normal colonic mucosa in individuals with CRN at sites distant from lesions. This is congruent with the theory of field cancerization, which proposes that cancer does not derive from a single isolated spot lesion. Instead, a larger area or “field” surrounding the cancer comprises cells that have acquired tumorigenic mutations that do not by themselves produce morphological changes but set the stage for cancer development [32].

It is well established that the injury‐inducible COX‐2 is associated with the development of CRN and CRC [7, 33]. This association is supported by the fact that treatment with aspirin, a nonselective COX‐inhibitor, at a low dose, reduces the risk of developing CRC [11, 12]. On the contrary, COX‐1 has mostly been associated with maintaining physiological functions and conditions during normal circumstances. The chemopreventive effects of aspirin, however, are likely due to an inhibition of both COX‐1 and COX‐2 synthesis of PGE_2_ and thereby reduced downstream inflammatory signaling and tumorigenic effects [5, 7, 12, 34]. In fact, a novel study by Yang et al. [35] showed that aspirin prevents CRC metastasis in a rodent model by inhibition of COX‐1 and thus its effect on platelet TXA_2_.

We found an increased COX‐activity in normal appearing colonic tissue from CRN patients, due to a combined contribution from both COX isoforms, when compared with biopsies from CTRLS. This suggests that both isoforms drive an abnormal COX‐PGE_2_ signaling cascade and likely carry tumorigenic properties. From our IHC staining of the human colonic epithelium, we observed that COX‐1 expression was dominantly restricted to tuft cells, whereas COX‐2 was expressed in absorptive cells [17, 22, 23]. Recently, Roulis et al. identified rare COX‐2 expressing pericryptal fibroblasts located near the stem cell zone and further demonstrated how constitutively synthesized PGE_2_, derived from these fibroblasts, could stimulate an expansion of reserve‐like stem cells. Ablation of COX‐2 in these fibroblasts significantly reduced tumorigenesis in a mouse model [21]. Single cell analysis showed that these cells also express COX‐1 [21]. We also noted COX‐1 expression in subepithelial cells near the stem cell zone and located near tuft cells [18]. These cells may very well represent the pericryptal fibroblasts that Roulis and colleagues identified. As tuft cells seem to be a considerable source of PGE_2_, it is tempting to speculate whether tuft cell derived PGE_2_ contributes to sporadic colonic tumorigenesis. Given the scarcity in general of experiments involving COX‐1 and CRN, there appears to be a large backlog of this type of experiments. This is particularly relevant considering the proposed involvement of COX‐1 in the development of CRC and its pronounced and selective expression in colonic tuft cells [22].

Along with the findings of an increased combined activity of COX‐1 and COX‐2, we also found an increase in mucosal content of PGE_2_ in CRN patients—a similar trend previously reported by Krishnan et al. [18, 36]. Meanwhile, it surprised us that the absolute concentrations of mucosal PGE_2_ were much greater than the concentration necessary for eliciting secretory responses. We previously speculated that this may be due to high intracellular storage of PGE_2_, which masked the extracellular concentrations when grinding biopsies for measurements. An alternative explanation could be that PGE_2_ synthesis following biopsy extraction was insufficiently inhibited. Considering the facts that the tissue is incubated only for a short period and the binding affinity (pK_i_) for indomethacin varies, it could be argued that the concentration of indomethacin should have been greater to maximize inhibition.

Overall, our observations suggest that normal appearing mucosa from patients with CRN is predisposed to neoplastic development through an overactive COX‐PGE_2_ axis. The underlying cause of the overactive COX‐PGE_2_ axis and the associated proinflammatory state remains incompletely understood. Potential contributing factors to chronic inflammation are dietary intake of omega‐6 short chain dienoic fatty acids such as linoleic acid (LA) and its long chain omega‐6 metabolites such as eicosatetraenoic acids (ETAs); examples are AA and its derivative PGE_2_. Conversely, the resolution of inflammation can be induced by dietary omega‐3 short chain trienoic polyunsaturated alpha‐linolenic acid (ALA) and its metabolites such as omega‐3 eicosapentaenoic acid (EPA) and docosahexaenoic acid (DHA). For instance, oxidation products of dietary LA have been implicated in the promotion of chronic inflammation and carcinogenesis [10]. Conversely, higher intake of ALA has been associated with a reduced risk of CRC in a Korean cohort [37]. However, while some studies have explored the relationship between dietary intake of fatty acids and CRC risk, findings provide only limited evidence supporting a protective association [38, 39, 40]. Interestingly, a recent meta‐analysis reported that omega‐3 fatty acid supplementation in patients with CRC is associated with improved clinical outcomes, including a reduction in postoperative infections and a shortened duration of hospital admissions [41].

A recent study by Soundararajan et al. [42], which examined both macroscopically visible lesions and adjacent normal‐appearing tissue, suggests that CRC is a result of disrupted lipid class switching, wherein the transition from dominant proinflammatory signaling to pro‐resolving lipid mediator signaling is impaired or fails to occur. Considering this, although our study focuses on differences between normal and affected colonic mucosa, the observed aberrant activity in the eicosanoid pathway in macroscopically normal tissue may reflect an early stage of defective lipid class switching.

Another potential target for CRC chemoprevention is the LOX signaling pathway. Notably, a few studies have suggested that there is a positive correlation between 5‐LOX expression levels and tumor size [34, 43, 44]. We observed increased expression of 5‐LOX, 12‐LOX, and 15‐LOX in normal appearing colonic mucosa from patients with CRN, suggesting that changes in the LOX signaling pathway occur early in tumorigenesis [9]. Both LOX enzymes, their leukotriene products, and receptors are under investigation as potential targets for chemoprevention and therapy. For example, the 5‐LOX inhibitor Zileuton has demonstrated the ability to reduce intestinal polyp formation and associated inflammation in animal models [45]. Montelukast, a leukotriene receptor agonist commonly used for asthma, is also under investigation for its potential chemopreventive effects, with a pilot clinical trial demonstrating a reduction in aberrant crypt foci, a biomarker for CRC [46]. Promising results have also emerged from studies targeting both COX and LOX pathways simultaneously [47]. To our knowledge, no clinical trials exist on 5‐LOX or combined COX/LOX inhibitors for the treatment nor chemoprevention of CRC.

We encourage further research in the field to also include the study of normal appearing colonic mucosa from CRN patients in addition to the well‐established methods in affected tissue samples such as transformed cell cultures and organoids. Including both will likely facilitate identifying connections between signaling pathways and human CRN. We believe this will enable the identification of early markers of CRN and pharmacological targets for prevention and treatment of CRN. Specifically, we noticed a need for further research regarding the role of COX‐1 in CRN/CRC. A compelling opportunity to further investigate this would be to examine the possible role of tuft cells in CRN/CRC. Also, a thorough examination of the leukotriene products and receptors as well as the CYP450 pathway in nonmalignant tissue is wanted.

## 5 Conclusions

The main take‐home messages from this review and from our laboratory are the following. Firstly, we believe that the study of normal *appearing* tissue (i.e., before lesions become macroscopically visible) is paramount in mapping out the very early changes leading to CRN development. Our experiments show that behind a normal appearance lie alterations in enzymes, receptors, transporters, and signaling pathways all associated with CRN and maybe even CRC. Potentially, these can be implemented as biomarkers for early disease detection/monitoring, or even therapeutic targets for new drugs.

Secondly, we believe that the role of COX‐1 in CRC development has been largely overlooked. Our findings suggest that both COX‐1 and COX‐2 are key drivers in the progression of CRC, especially during the early stages of the disease. Consequently, we advocate for a more thorough examination of the contribution of COX‐1 to CRC pathogenesis.

## Author Contributions

**Ulrike Ries Feddersen:** methodology, investigation, project administration, writing – original draft, writing – review and editing. **Sebastian Kjærgaard Hendel:** investigation, visualization, project administration, writing – original draft, writing – review and editing. **Victoria Hellen Berner‐Hansen:** investigation, writing – original draft, writing – review and editing. **Simon Veedfald:** investigation, writing – review and editing. **Mark Berner‐Hansen:** conceptualization, investigation, supervision, writing – review and editing. **Niels Bindslev:** conceptualization, investigation, supervision, project administration, writing – original draft, writing – review and editing.

## Conflicts of Interest

The authors declare no conflicts of interest. Mark Berner‐Hansen is also an employee of Zealand Pharma. This affiliation is unrelated to this paper.

## Data Availability

Data sharing is not applicable to this article as no new data were created or analyzed in this study.
